# Effects of Maternal Anemia Affecting Fetal Outcomes: A Narrative Review

**DOI:** 10.7759/cureus.64800

**Published:** 2024-07-18

**Authors:** Madhura A Kharate, Sonali G Choudhari

**Affiliations:** 1 Department of Community Medicine, Jawaharlal Nehru Medical College, Datta Meghe Institute of Higher Education and Research, Wardha, IND

**Keywords:** treatment, prenatal mortality, neonatal mortality, maternal mortality, fetal outcomes, maternal anemia, pregnancy, anemia

## Abstract

This review’s main objective was to assess the obstacles to anemia prevention, as well as the attitudes and behaviors of anemic women toward their condition. Since iron is crucial for neurodevelopment, iron deficiency anemia (IDA) accounts for the majority of pregnant mothers having anemia. In India and other developing countries, anemia is a serious health problem. More than half of pregnant women have anemia. The search strategy was conducted in PubMed. Few of the articles were searched without using MeSH terms. Strong correlations between mothers' anemia and that of their offspring point to intergenerational anemia with lasting consequences. Children who were underweight at birth and those who were malnourished had a higher risk of having anemia. Clinicians usually evaluate anemia, and the criteria for determining the cause of anemia are outlined in this brief review.

## Introduction and background

More than 800 million women and young children suffer from anemia, which is a serious global health issue. The World Health Organization (WHO) reports that 29% of all women and 38% of pregnant women are anemic globally [1]. The WHO defines anemia as a condition where a pregnant woman’s hemoglobin is less than 11 g/dL [2]. The primary cause of iron deficiency anemia (IDA) often stems from continuous blood loss, typically from the uterine or gastrointestinal tract, in affluent nations [3]. A precious experience, pregnancy can affect both the mother's health and the health of future generations. It is generally known that nutrition is important for the health of mothers and children and that a balanced diet during infancy lays the groundwork for long-term health [4]. Pregnancy anemia should be diagnosed as soon as possible to avoid difficulties for maternal and child health [5]. The factors contributing to anemia in pregnancy include poverty, young mothers, and inadequate iron intake, which are comparable worldwide [4]. Micronutrient deficiencies are characterized as a person's diet not providing enough of the required daily levels of vitamins and minerals for proper health, growth, and development [6]. Anemia during pregnancy has a significant impact on world health. Due to the various causes, its effects vary from one place to another. Additionally, nothing is known about the relationships between anemia in the first trimester of pregnancy and future outcomes [4]. One of the major global public health concerns and a main cause of disability is anemia [7].

Anemia continues to be one of the major causes of frailty and one of the most important global health problems anywhere in the world. This is due to the enormous importance of pregnancy in both industrialized and non-industrialized nations. According to a recent WHO report, 32 million pregnant women worldwide are anemic, which is around 38% of the population. Nevertheless, the reasons for the rate typically highlight global inconsistencies [7]. Premature birth, low birth weight (LBW), abortion, delayed psychomotor development, impairment of cognitive function, and worse scores on IQ tests for newborns are a few of these factors, and they all have an impact on the children’s later lives. Hemoglobin levels under 11 g/dL are considered anemic by the WHO. Preterm babies need lengthy hospital stays and are more likely to experience negative outcomes such as respiratory problems, neurodevelopmental aftereffects, necrotizing enterocolitis, feeding issues, blindness, deafness, and intraventricular hemorrhage [8]. Malnutrition, parasites, chronic conditions, and malaria all contribute to IDA. In developing countries, more than two-thirds of pregnant mothers have anemia, and iron deficiency is responsible for 95% of cases. About 84% of women experience an iron deficit during the first postpartum week.

Women in underdeveloped countries typically experience anemia during pregnancy, suggesting that prior iron levels are often scant and that intellectual changes accelerated by pregnancy are insufficient to meet the growing demands. Pregnancy iron supplementation is now a common and accepted procedure to prevent the onset of IDA. The effectiveness of treatment for anemia in pregnancy was reviewed in light of the aforementioned information. Additionally, flaws were found, and suggestions for advancement were provided [9]. This review is undertaken with the objective of assessing the obstacles to anemia prevention and how anemic women perceive and treat their disease.

## Review

**Figure 1:**
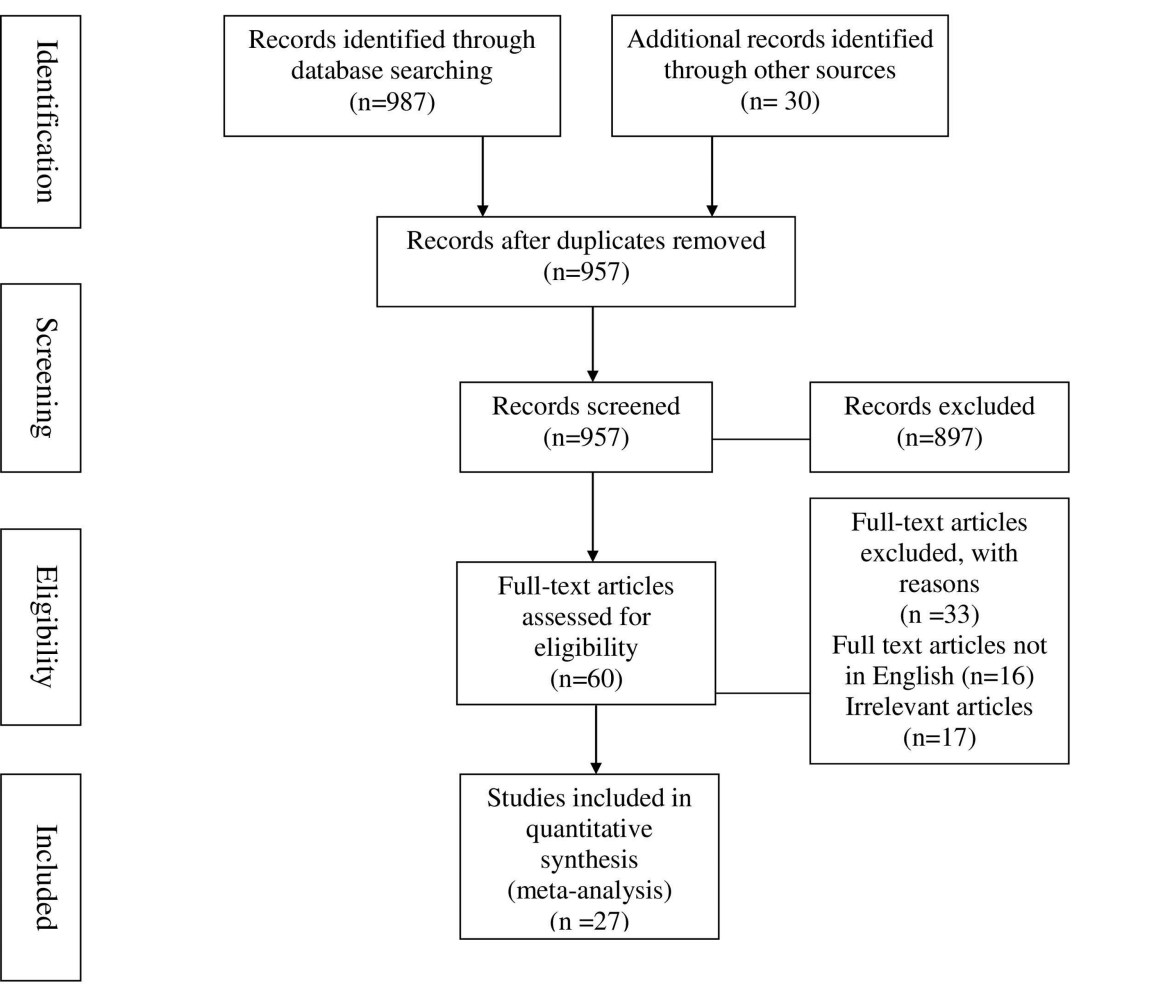
Exclusion and inclusion criteria of the study

This review is based on the determinants affecting anemic mothers. The references used in this article are sourced from sites such as PubMed, WHO, NCBI (National Center for Biotechnology Information), and others. The search terms used include (((anemia AND pregnant women) AND neonatal mortality) AND low birth weight) AND antenatal mortality AND perinatal mortality AND maternal mortality AND (India [MeSH Terms]). Of the total 60 studies initially assessed for eligibility, 27 were analyzed in this review. The aim of this article is to examine whether pregnant women suffering from anemia are treated appropriately and to address issues related to child mortality, LBW, neonatal mortality, and maternal mortality. Figure 1 shows the inclusion and exclusion criteria for the study.

Discussion

The WHO has declared that IDA is the most severe nutritional deficiency of the 21st century worldwide, emphasizing that women are particularly susceptible. If overlooked and handled incorrectly, such a situation might have catastrophic impacts on entire communities and serious ramifications [10]. IDA has been identified by the WHO as the most critical nutritional deficiency globally, with particular emphasis on the disproportionate risk faced by women. The increased demands for iron during pregnancy - due to the growth of the placenta, the developing fetus, and the mother's red blood cells - underscore the heightened significance of addressing this issue [10]. If such a scenario is overlooked or handled improperly, it can have devastating consequences for entire populations and significant repercussions. The maternal and fetal outcomes of the case and control groups, including hemoglobin levels, were comparable [11]. An approach for addressing IDA in pregnancy and the postpartum period is proposed, supported by insights from various prospective randomized trials. Anemia and iron deficiency were common early in pregnancy and were linked to a higher risk of unfavorable pregnancy and baby outcomes [12].

To minimize or eliminate the necessity for blood transfusions and efficiently replenish iron levels, the use of intravenous iron is becoming more prevalent [13]. A cohort study carried out in rural Bihar, India, reveals a significant correlation between the hemoglobin levels of pregnant women and the hemoglobin levels observed in their offspring from 22 to 32 months after birth [9]. However, we did not observe any differences among women experiencing stillbirth; those with mild anemia contribute to the rate, showing only lower birth weights and a higher likelihood of premature birth. In terms of maternal outcomes, we found that women with severe anemia had an increased likelihood of postpartum hemorrhage and a decreased chance of undergoing cesarean delivery. Although the differences in maternal mortality did not reach statistical significance, the mortality rate was highest among women with severe anemia compared to other groups. Mild anemia showed no adverse effects [8]. We could not find any links between mild anemia and poor outcomes, but low-birth-weight babies were more likely to be delivered by mothers who were moderately anemic [14]. There have not been any major side events reported from the few studies done on the use of iron polymaltose throughout pregnancy and the neonatal stage. Only the intramuscular (IM) route is used for its administration [15]. The study excluded patients who had another known cause of anemia, such as vitamin B12 deficiency, thalassemia, or sickle cell disease, as well as those who were actively bleeding when admitted for labor and delivery [16].

**Figure 2:**
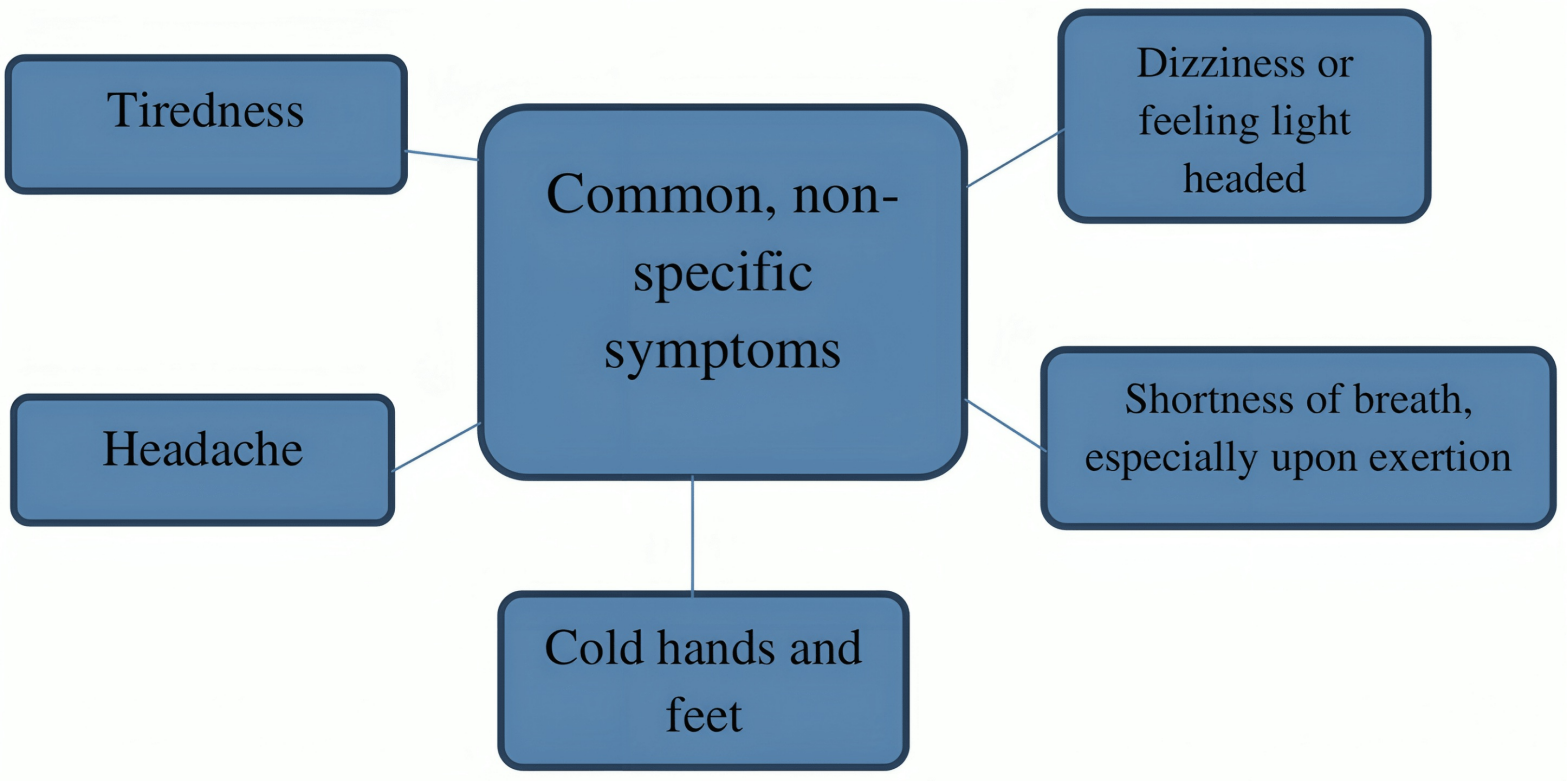
Symptoms of anemia

The results of the current review indicate that iron supplementation in sickle cell pregnancy should only be justified after determining whether or not the mother's iron stores are adequate, as the levels are more evenly distributed than in the control group. Before beginning iron prophylaxis, guidelines also advocate evaluating the iron status of pregnant women [17]. Iron supplementation in the form of ferrous bis-glycinate, with folinic acid and multivitamins, demonstrates enhanced improvements in hematological parameters, iron absorption, quality of life, and birth weight among iron-deficient pregnant women, as compared to ferrous fumarate [18]. Maternal iron insufficiency can significantly impact fetal health, highlighting the importance of addressing this condition. In addition to increasing the risk of fetal death, LBW, and preterm birth, iron deficiency during pregnancy is linked to long-term cardiovascular, metabolic, neurobehavioral, and immunological issues in afflicted offspring [19]. Problems with sleep during pregnancy may include little sleep and poor sleep quality [20]. Figure 2 shows the symptoms of anemia.

Detecting and addressing IDA during pregnancy is crucial due to potential complications, such as premature birth, developmental delays in the womb, placental problems, reduced iron storage in newborns, decreased maternal blood stockpiles during childbirth, the need for transfusions in cases of significant hemorrhage, heart failure, indications of anemia, an extended hospital stay, diminished production of maternal milk, and exhaustion of maternal iron concentrations [15]. The key element in diagnosing iron-deficiency anemia is laboratory testing. Traditional laboratory findings indicative of IDA include a decrease in hemoglobin, an increase in total iron-binding capacity, an elevation in serum ferritin levels, and a reduction in serum iron concentration.

However, even if the baseline number is normal, it is always advisable to monitor hemoglobin every trimester because there is always a chance that the need for iron will increase and an iron deficiency will emerge. A low maternal hemoglobin level can cause fetal issues, including mortality; hence, the hemoglobin concentration during birth is crucial [15]. Even in the absence of anemia, patients with newly diagnosed anemia, as well as those being evaluated for fatigue or restless leg syndrome, should have iron assays done. Latent iron deficiency (LID), which some people may have despite not having overt anemia, is characterized by low iron storage [21].

When pregnant patients with anemia present, a history of menorrhagia, pre-pregnancy hemoglobin levels, frequent childbirths, worm passing, and gastrointestinal blood loss should be obtained. We do not advise stool tests for occult blood, worm infestation, gastrointestinal endoscopies, or celiac disease screening unless expressly indicated because the cause of IDA in pregnancy often reflects an imbalance between the supply and demand of iron [22]. Table 1 shows how the diet should be managed to avoid anemia during pregnancy, which can lead to maternal mortality, LBW, and other complications.

**Table 1:** Self-care healthy diet to be consumed to avoid anemia during pregnancy

Iron-rich foods	Foods rich in vitamin C	Foods to avoid
Lean red meats, fish, poultry, legumes (e.g., lentils and beans), fortified cereals, and dark green leafy vegetables	Foods rich in vitamin C (such as fruits and vegetables), which help the body absorb iron	Foods that slow down iron absorption when consuming iron-rich foods, such as bran in cereals (whole wheat flour, oats), tea, coffee, cocoa, and calcium

The severity of the hemoglobin level and the woman's overall health will determine the course of treatment. Oral iron therapy is typically advised for mild cases of anemia; however, it can be shifted to intravenous iron treatment for patients with moderate or severe anemia, or for those who cannot tolerate oral iron supplements. Once the target values have been reached, oral iron preparations can be used for maintenance therapy [15].

Child health outcomes

LBW

Generally, reduced hemoglobin levels measured at various time points were associated with an increased risk of LBW as follows: preconception (odds ratio (OR): 1.72, 95% confidence interval (CI): 1.31-2.26), first trimester (OR: 1.23, 95% CI: 1.07-1.41), second trimester (OR: 1.14, 95% CI: 0.78-1.68), and third trimester (OR: 1.65, 95% CI: 1.39-1.96). The only trimester where this association was not statistically significant was the second. Although this estimate was based on sparse data, low hemoglobin levels during the prejudgment interval had the strongest association with higher risks of LBW [7].

Overall, the estimation of low maternal hemoglobin was linked to a higher risk of LBW (OR: 1.42, 95% CI: 1.31-1.55) in a meta-analysis of all pregnancy studies (n = 36). Although there was a similar trend, high maternal hemoglobin (n = 5) was not significantly linked with LBW (OR: 1.80, 95% CI: 0.86-3.77) [23]. LBW, defined as a weight of less than 2500 g at birth, is associated with increased risks of infant death, stunting, developmental delays, and metabolic illnesses that manifest in adulthood [24]. LBW infants weigh less than 2500 g, whereas high-birth-weight infants weigh more than 2500 g (normal birth weight).

Prenatal Mortality

Data on the timing of hemoglobin assessment were scarce. There was a persistent trend toward higher maternal mortality linked to decreased maternal hemoglobin throughout gestation, even though trimester-specific data were not statistically significant. The connection between increased maternal hemoglobin and prenatal mortality lacked sufficient data. When data from various time points, including studies with unspecified timing and cutoffs, were combined, there was an increased risk of prenatal mortality associated with low maternal hemoglobin (n = 11). The OR with a 95% CI was 1.73 (1.32-2.26) [23].

Neonatal Mortality

The unit with grievous anemia had a significantly higher probability of infant mortality at 28 days (the primary outcome). Grievous iron deficiency was also strongly related to an increased risk of LBW (2500 g), very LBW (1500 g), preterm birth, and neonatal mortality [25]. Twenty-nine pregnancies resulted in a total of 25 live births (89.3%). Ten (35.7%, n = 28) of the study population experienced preterm births. Out of 25 live deliveries, 10 (or 40%) had birth weights under 2.5 kg and were admitted to the NICU. Three (12%) of the 25 live babies had an APGAR score of 7 at the first minute after birth.

Maternal Mortality

In the study population, there were 29 maternal deaths, 20 of which were caused by severe anemia. One fatality had moderate/severe anemia as the primary cause, while 19 deaths had it as a coexisting disease. Out of the 29 fatalities, eight were anemic and underweight, 19 were anemic alone, one was underweight alone, and two were overweight or obese. Gush (n = 9, or 31%), toxemia (n = 7, or 24%), infirmity (n = 6, or 21%), and misplacement (n = 7, or 22%) were the main causes of maternal fatalities [25]. Maternal death and other unfavorable maternal outcomes, known to increase maternal mortality, were observed in women with antepartum anemia. In order to improve the mother's health and perhaps lower maternal morbidity and mortality, anemia must be identified and treated during the antepartum period [26].

Association Between Childhood Anemia, Early Skills, and Pregnant Anemia

A 0.17 g/dL increase in the mother's hemoglobin level in her offspring was linked to an upsurge of 1 g/dL in her hemoglobin during gestation. Hemoglobin levels were 0.20 g/dL lower among kids delivered to mothers who were slightly anemic than in infants delivered to mothers who were not anemic, and a greater than double as a large factor for babies delivered to women who were moderately or severely anemic. Mothers' current hemoglobin levels and those of their offspring consistently correlated positively, with a 1% significance level. Evaluating the U-shaped bond, it was found that there was a productive but diminishing interrelationship between pregnant hemoglobin and infant hemoglobin, achieving extraordinary results at the 5% level [9].

Neurophysiological Development

In four types of research, neurophysiological evaluations were used. Positive correlations between infant responses and iron levels were found in all four investigations. However, given that none of the studies were of good quality, these findings should be regarded with caution [27].

## Conclusions

A human rights-based approach to universal health calls for the provision of high-quality care not only before and after childbirth but also during pregnancy and labor. Our findings provide insight into the impacts of iron deficiency based on gestational age and the appropriate timing of taking iron supplements during pregnancy. In several specific indications, such as intolerance, poor patient compliance, inadequate treatment response, and longer therapy duration, these preparations are superior to oral iron preparations. Consideration should be given to a worldwide, all-encompassing IDA management strategy that offers a variety of evidence-based treatment options and addresses regional concerns. However, in developing countries where IDA is pervasive, resource constraints are a major issue.
